# Behavioral Phenotype in the TgF344-AD Rat Model of Alzheimer’s Disease

**DOI:** 10.3389/fnins.2020.00601

**Published:** 2020-06-16

**Authors:** Rachel Michelle Saré, Spencer K. Cooke, Leland Krych, Patricia M. Zerfas, Robert M. Cohen, Carolyn Beebe Smith

**Affiliations:** ^1^Section on Neuroadaptation and Protein Metabolism, National Institute of Mental Health, National Institutes of Health, Department of Health and Human Services, Bethesda, MD, United States; ^2^Diagnostic and Research Services, Office of Research Services, National Institutes of Health, Department of Health and Human Services, Bethesda, MD, United States; ^3^Department of Psychiatry and Behavioral Sciences, Emory University School of Medicine, Atlanta, GA, United States

**Keywords:** Alzheimer’s disease, amyloid-β, actigraphy, hyposmia, learning

## Abstract

Alzheimer’s disease (AD) is a progressive neurodegenerative disease resulting in cognitive decline. A unique rat model, TgF344-AD, recapitulates pathological hallmarks of AD. We used a longitudinal design to address the timing of expression of behavioral phenotypes in male and female TgF344-AD rats. In both sexes, we confirmed an age-dependent buildup of amyloid-β. In the open field, female, but not male, TgF344-AD rats were hypoactive at 6 and 12 months of age but at 18 months the two genotypes were similar in levels of activity response. Both male and female TgF344-AD rats had a deficit in performance on a learning and memory task. Male TgF344-AD, but not female, rats had evidence of hyposmia regardless of age. Rest-activity rhythms followed the typical active/inactive phase in all rats regardless of genotype or age. In males, home cage activity was similar across age and genotype; in females, regardless of genotype animals were less active as they aged. These changes highlight some behavioral markers of disease in the rat model. Early markers of disease may be important in early diagnosis and assessment of efficacy when treatment becomes available.

## Introduction

Alzheimer’s disease (AD) is a neurodegenerative disorder of aging characterized by progressive cognitive decline and, ultimately, dementia. This disorder affects millions of people and is more prevalent in females than in males (Vina and Lloret, 2010). AD has distinctive pathological characteristics including neurofibrillary tangles (composed of hyperphosphorylated tau) and the accumulation of amyloid-β plaques. Whereas neurofibrillary tangles and amyloid-β plaques have been studied as potential targets for AD therapeutics, no effective treatment target has been established (Kumar et al., 2015).

It is generally accepted that AD pathology begins decades before symptoms of cognitive decline are apparent (Sperling et al., 2014). For a potential treatment to be effective, it is essential that the disease is diagnosed early in this “preclinical” window. In our study we characterized an animal model of AD over the course of the aging process from maturity to senescence to uncover additional behavioral phenotypes that might be markers of the appearance of this degenerative disorder. We used the TgF344-AD rat model developed in 2013 on a Fisher344 background (Cohen et al., 2013). This transgenic model contains two mutations implicated in AD: overexpressing human amyloid precursor protein (APPsw) and presenilin 1 (PSE1E9). This model recapitulates the pathological changes in AD including neurofibrillary tangles, amyloid-β plaques, neuronal loss, and gliosis. Moreover, the TgF344-AD rat has demonstrable age-dependent indications of declining memory function (Cohen et al., 2013; Do Carmo and Cuello, 2013) and cognitive impairments (Berkowitz et al., 2018; Munoz-Moreno et al., 2018).

Our findings confirm an age-dependent accumulation of amyloid-β accompanied by a deficit in learning and memory. In addition, we found several behavioral changes that may precede cognitive decline. Female TgF344-AD rats were hypoactive in the open field at 6 months of age. Male TgF344-AD rats exhibited evidence of hyposmia at 6 months of age. These data suggest novel phenotypes that may be used in conjunction with other diagnostic markers to aid in early disease identification.

## Materials and Methods

### Animals

All procedures were approved by the National Institute of Mental Health Animal Care and Use Committee and followed the National Institutes of Health Guidelines on the Care and Use of Animals. Tg344-AD^+/–^ breeder pairs (wild type females and heterozygous males) were obtained from Emory University (Atlanta, GA, United States) and mated to provide heterozygous and control male and female offspring. Rats were group housed with food and water *ad libitum* and maintained in a centralized facility with a normal 12-h light dark cycle. Behavior testing was performed in the light phase between 9:00 AM and 5:00 PM. Tail snips were taken at weaning from each animal for genotyping as previously described (Cohen et al., 2013).

Behavior analyses (open field, buried food task, and reward alternating T-maze) were performed in a longitudinal study. Home cage activity assessment was analyzed in a cross-sectional study. This change in design was necessitated by limitations in equipment availability for home cage activity assessment. For analysis of home cage activity, each animal was assigned to one age category in an unbiased manner.

### Behavior Testing

Prior to behavior testing, animals were habituated to handling for 14 days. Seven days prior to testing, animals were habituated to the reward (Froot Loops^TM^) in their home cages. Prior to all behavior testing, animals were habituated to the test room for 30 min in their home cages. Animals were separated into two cohorts for testing. Both cohorts underwent behavioral testing as follows: open field testing, buried food task, then reward alternating T-maze. The first cohort had 26 animals and an average of 8 days between open field testing and the buried food task and an average of 26 days between buried food task and reward alternating T-maze testing. The second cohort had 10 animals and waited an average of 23 days between open field testing and buried food task and 4 days between buried food task and reward alternating T-maze testing.

### Open Field Test

We assessed activity by means of a standard open field apparatus consisting of a clear Plexiglass chamber measuring 40.6 × 40.6 × 38 cm (Coulbourn Instruments, Whitehall, PA, United States). The test animal was placed in the center of the apparatus and allowed to explore for a 60 min period. Infrared beams were used to detect the animal’s movements. We determined total horizontal distance moved for each animal in 10 min epochs.

### Buried Food Task

We used an adapted version of the buried food task to measure olfaction (Yang and Crawley, 2009). Prior to testing, the animal was food-deprived 18–24 h and habituated to the testing cage (a standard cage with 3 cm of bedding) for 5 min. Following habituation, the animal was transferred to a temporary holding cage while the reward was buried in a randomly selected location 1 cm below the surface of the bedding in the testing cage. The animal was then returned to the opposite side of the test cage from the buried reward and the latency to find the reward was determined (15 min maximum). Of the 34 animals tested, 16 reached the time out criterion: eight at 6 months, three at 12 months and 11 at 18 months of age. These animals were assigned the maximum time (900 s).

### Reward Alternating T-Maze

We used reward alternation in the T-maze as a measure of working memory (Deacon and Rawlins, 2006). Rats were habituated to the maze with a reward placed at both ends daily for 7 days with 10 min of free exploration. Animals were food-restricted 18–24 h before and during the 3-day testing phase. Testing consisted of two trials per day (with an inter-trial interval of 1 h). A reward was placed at both ends of the T-maze, but one arm (randomly chosen) was blocked. This was defined as the choice arm. The unblocked arm was defined as the sample arm. The animal was placed at the start of the maze and allowed to explore until it found and consumed the reward in the sample arm (maximum of 10 min allowed). The animal was then returned to the start of the maze and the barrier was removed from the choice arm. The number of times the animal correctly chose the choice arm was recorded as a percent correct over total choices. If the correct arm was selected, the animal could consume the reward before being returned to the home cage.

### Home Cage Activity

We used the Comprehensive Laboratory Animal Monitoring System and Oxymax software (Columbus Instruments, Columbus, OH, United States) to monitor activity in the home cage over a 24-h period. The home cage containing the test animal was placed in the recording apparatus in which photobeams were used to detect movement in the *y* axis. Movement was recorded every 10 s and binned in 1-h epochs. Animals were allowed 2 days of habituation to the recording environment. Activity was recorded in the inactive (light) and active (dark) phases across a 24-h period.

### Immunohistochemistry

In a separate group of 14 TgF344-AD animals, we stained for amyloid-β with immunohistochemistry. We analyzed (one male and three females at 4–6 months, one female at 12 months, and four males and five females at 24–25 months). Given the low number of males at 6 and 12 months of age, we combined sexes for analysis of age-effects of amyloid plaque buildup. Brain tissue was embedded in paraffin and sections 5 μm in thickness were prepared. Sections were taken at the level of hippocampus, cortex, and cerebellum. The antigen was retrieved with citrate buffer, pH 6.0 for 20 min at 95°C in a pressure cooker and allowed to cool to room temperature. The slides were rinsed in TBST followed by distilled water, treated with SNIPER (Biocare, Pacheco, CA, United States) for 10 min, and blocked with serum free protein block from DAKO (Cat No X0909) for 5 min. Slides were incubated with amyloid-β antibody (Clone DE2) (1:100, Millipore, Temecula, CA) for 45 min. The slides were rinsed two times with TBST, incubated with DAKO Envision mouse peroxidase (DAKO, Carpentaria, CA, United States) for 30 min followed by 3,3′-diaminobenzidine (DAB+; DAKO) for 7 min, and counterstained with hematoxylin. For a negative control, the primary antibody was replaced with mouse IgG at the same concentration. Amyloid-β plaques appeared as brown stellate structures 5–80 μm in diameter. We measured amyloid-β positive plaque area in four brain regions defined by reference to an atlas of the rat brain (Paxinos and Watson, 1986). We expressed results as % of total regional area in the field of view.

### Statistical Analyses

Data are expressed as means ± standard errors of the mean (SEM). Results of behavior testing were analyzed by means of repeated measures ANOVA (RMANOVA) with SPSS version 21 (IBM, Armonk, NY, United States) with genotype as the between subjects variable and age as a within subjects variable. Males and females were analyzed separately. Open field testing was analyzed with epoch as an additional within subjects variable. Since home cage activity was studied in a cross-sectional design, both genotype and age were between subjects variables and epoch was a within subjects variable. *P*-values < 0.05 were considered statistically significant and are marked with a “^∗^,” and *p*-values 0.10 ≥ *p* > 0.05 are indicated with a “†.”

## Results

### Survival

Rats were bred in-house, such that control and TgF344-AD rats were littermates. For these behavioral studies, we began with 20 control (14 male and 6 female) and 22 TgF344-AD (6 male and 16 female) rats, and we include data from animals [15 controls (nine male, six female) and 19 TgF344-AD (15 female, 4 male)] that were tested at all three ages, 6, 12, and 18 months. Of the original cohorts, five control (all male) and three TgF344-AD (two male and one female) rats died before all the tests at 18 months. The cause of death for the TgF344-AD rats was pituitary hemorrhage in one male rat and testicular cancer in another. The cause of death for the female TgF344-AD rat was not determined. The cause of death for three of the control males was as follows: one died during surgical removal of an islet tumor, one was euthanized because of a large subcutaneous tumor on a forelimb, and one from testicular cancer. The cause of death in the other two animals could not be determined. Of our original cohort, survival at 6 and 12 months of age was 100% for both groups. At 18 months of age survival was 75 and 91% for control and TgF344-AD rats, respectively. For control males and females at 18 months of age, survival was 64 and 100%, respectively. For TgF344-AD rats, survival at 18 months of age was 67 and 94% in males and females, respectively.

### T-Maze

**FIGURE 1:**
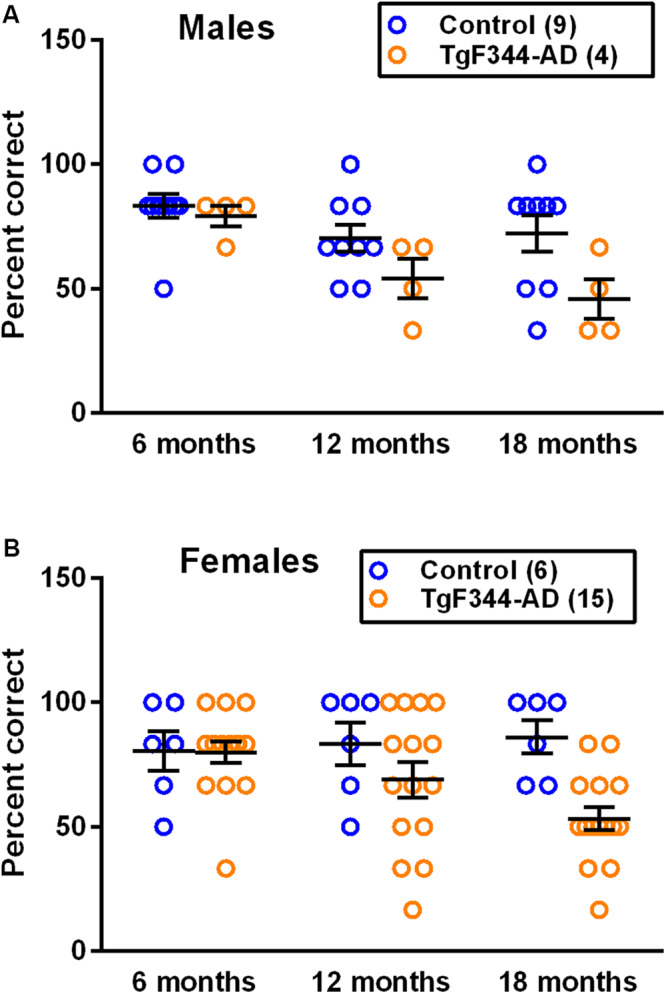
Performance on the reward-alternation T-maze in **(A)** male and **(B)** female control and TgF344-AD rats. Each point is the percent correct alternations, and the lines represent the mean ± SEM for the number of male and female control and AD rats indicated in parentheses. The main effect of genotype was statistically significant in both males (*p* = 0.015) and females (*p* = 0.024). The main effect of age was statistically significant in males (*p* = 0.014) but not in females. Results indicate a decreased performance in AD rats in both sexes. In males, performance of both genotypes declines with age.

We used the alternating T-maze task to examine learning and memory. We found statistically significant main effects of genotype in both male (Table 1 and Figure 1A) and female rats (Table 2 and Figure 1B) on the reward-alternation T-maze task indicating that, regardless of age, performance was worse in the TgF344-AD rats compared to controls. In males, but not females, the effect of age was also statistically significant indicating that regardless of genotype performance declined as males aged (Figure 1A).

**TABLE 1:** Repeated measures ANOVA results behavior testing in males.

Behavior	Interaction	Main effect	*F*_(df, error)_ value	*P*-value	Cohen’s *f*^2^
**T-maze**					
Fraction correct	Genotype × age		*F*_(__2_,_22__)_ = 1.124	0.343	0.103
		Genotype	*F*_(__1_,_11__)_ = 8.276	0.015*	0.751
		Age	*F*_(__2_,_22__)_ = 5.235	0.014*	0.475
**Buried food**					
Latency	Genotype × age		*F*_(__1__.__8_,_20__)_ = 1.012	0.375	0.092
		Genotype	*F*_(__1_,_11__)_ = 4.992	0.047*	0.453
		Age	*F*_(__1__.__8_,_20__)_ = 1.659	0.216	0.151
**Open field**					
Total distance moved	Genotype × age × epoch		*F*_(__6__.__6_,_73__)_ = 0.947	0.443	0.086
	Age × epoch		*F*_6__.__6_,_73__)_ = 1.520	0.177	0.138
	Genotype × epoch		*F*_(__4__.__9_,_54__)_ = 1.837	0.122	0.167
	Genotype × age		*F*_(__2_,_22__)_ = 1.271	0.300	0.116
		Genotype	*F*_(__1_,_11__)_ = 0.348	0.567	0.032
		Age	*F*_(__2_,_22__)_ = 2.805	0.082^†^	0.255
		Epoch	*F*_(__4__.__9_,_54__)_ = 23.495	0.001*	2.135
**Home cage activity**					
	Genotype × age × epoch		*F*_(__31__.__4_,_424__)_ = 1.142	0.277	0.071
	Age × epoch		*F*_(__31__.__4_,_424__)_ = 1.004	0.463	0.066
	Genotype × epoch		*F*_(__15__.__7_,_424__)_ = 0.923	0.542	0.031
	Genotype × age		*F*_(__2_,_27__)_ = 1.589	0.223	0.140
		Genotype	*F*_(__1_,_27__)_ = 0.001	0.979	0.029
		Age	*F*_(__2_,_27__)_ = 0.638	0.536	0.055
		Epoch	*F*_(__15__.__7_,_424__)_ = 21.287	<0.001*	0.524

**TABLE 2:** Repeated measures ANOVA results behavior testing in females.

Behavior	Interaction	Main effect	*F*_(__df, error__)_ value	*P*-value	Cohen’s *f*^2^
**T-maze**					
Fraction Correct	Genotype × age		*F*_(__1__.__66_,_31__)_ = 3.005	0.073^†^	0.159
		Genotype	*F*_(__1_,_19__)_ = 5.989	0.024*	0.316
		Age	*F*_(__1__.__66_,_31__)_ = 1.301	0.280	0.068
**Buried food**					
Latency	Genotype × age		*F*_(__2_,_38__)_ = 2.464	0.099^†^	0.130
		Genotype	*F*_(__1_,_19__)_ = 2.287	0.147	0.120
		Age	*F*_(__2_,_38__)_ = 2.450	0.100^†^	0.129
**Open field**					
Total distance moved	Genotype × age × epoch		*F*_(__9_,_174__)_ = 0.893	0.534	0.047
	Age × epoch		*F*_(__9_,_174__)_ = 3.614	<0.001*	0.190
	Genotype × epoch		*F*_(__4__.__3_,_81__.__7__)_ = 1.020	0.405	0.054
	Genotype × age		*F*_(__2_,_37__.__7__)_ = 4.886	0.013*	0.258
		Genotype	*F*_(__1_,_19__)_ = 8.535	0.009*	0.449
		Age	*F*_(__2_,_37__.__7__)_ = 23.081	<0.001*	1.212
		Epoch	*F*_(__4__.__3_,_81__.__7__)_ = 78.147	<0.001*	4.102
**Home cage activity**					
	Genotype × age × epoch		*F*_(__21_,_327__)_ = 1.399	0.115	0.096
	Age × epoch		*F*_(__21_,_327__)_ = 1.861	0.013*	0.121
	Genotype × epoch		*F*_(__10__.__6_,_327__)_ = 1.297	0.227	0.056
	Genotype × age		*F*_(__2_,_31__)_ = 1.131	0.336	0.073
		Genotype	*F*_(__1_,_31__)_ = 1.090	0.305	0.035
		Age	*F*_(__2_,_31__)_ = 8.733	0.001*	0.563
		Epoch	*F*_(__10__.__6_,_327__)_ = 29.077	<0.001*	1.475

### Buried Food Task

**FIGURE 2:**
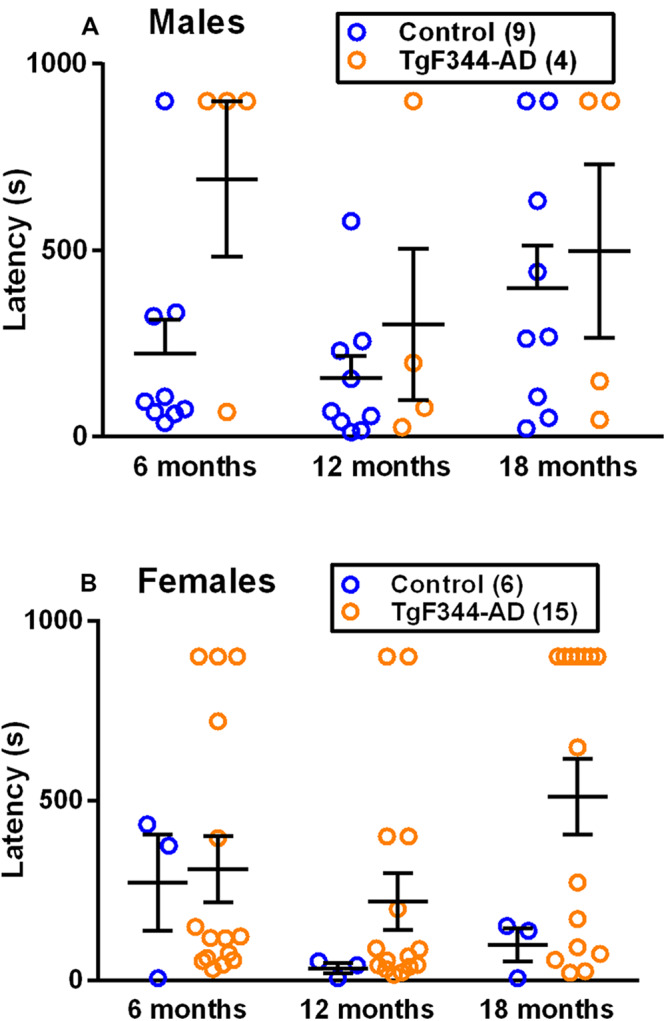
Performance on the buried food task in **(A)** male and **(B)** female TgF344-AD and control rats. Each point is the latency to find the buried food, and the lines represent the mean ± SEM for the number of male and female control and AD rats indicated in parentheses. The main effect of genotype was statistically significant (*p* = 0.047) in males but not in females. Latency to find the buried food was longer in male AD rats compared to controls.

Hyposmia is common in patients with AD and worsens with progression of the disease, but the timing of the initial presentation of this symptom is not known (Serby et al., 1991). To study hyposmia and the development of this phenotype, we performed the buried food task in a longitudinal study. We found a statistically significant main effect of genotype (*p* = 0.047) in males (Table 1 and Figure 2A), but not in females (Table 2 and Figure 2B). These results indicate that in males, regardless of age, the latency to locate the buried food is longer in TgF344-AD rats compared to controls.

### Activity in the Open Field

**FIGURE 3:**
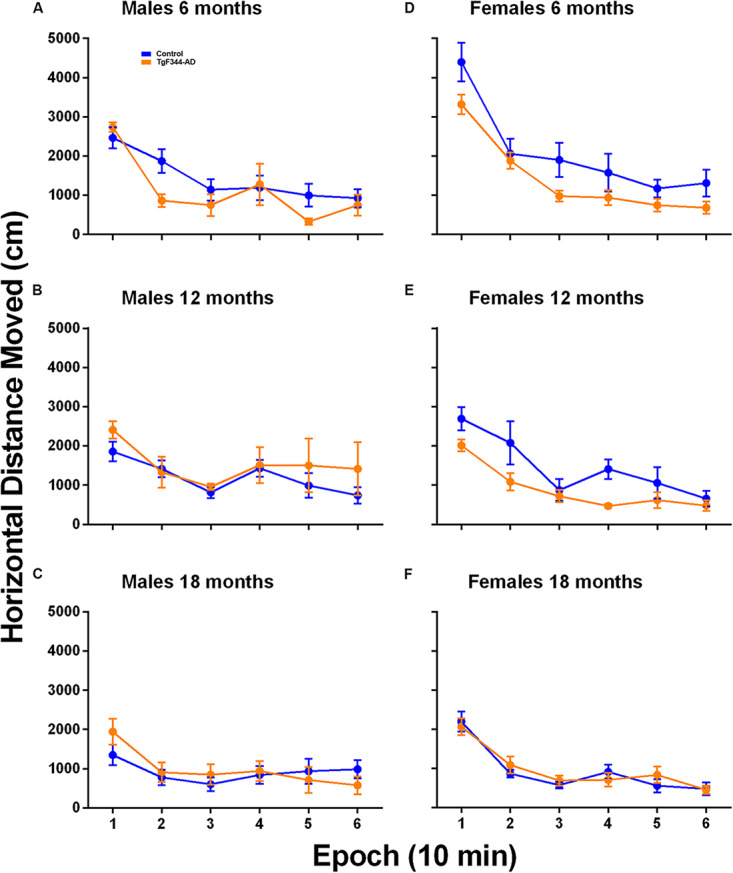
Open field activity in **(A–C)** male and **(D–F)** female TgF344-AD and control rats at **(A,D)** 6 months, **(B,E)** 12 months, and **(C,F)** 18 months of age. Each point is the mean ± SEM in nine male controls, five male TgF344-AD, six female control and 15 female TgF344-AD rats. For males, only the main effect of epoch was statistically significant (*p* < 0.001) indicating habituation over the 60 min testing period regardless of either age or genotype. For females, the age × epoch (*p* < 0.001) interaction was statistically significant indicating that regardless of genotype habituation decreased with increasing age likely due to decreased responsivity to the environment in the 12- and 18-month old animals. Moreover, in females, the genotype × age (*p* = 0.013) interaction was statistically significant, indicating that in the 6 and 12 month old groups, AD rats were hypoactive compared with controls; activity levels were similar in the two genotypes at 18 months of age.

To determine activity levels in response to a novel environment, we performed open field testing for 1 h. In males, only the main effect of epoch was statistically significant indicating that animals, regardless of age or genotype, showed habituation to the arena (Table 1 and Figures 3A–C). In females, the age × epoch interaction was statistically significant (Table 2 and Figures 3D–F). In the first three epochs, animals at 6 months of age moved more in response to the novel environment compared with older rats suggesting that the younger animals were more reactive than older animals (Supplementary Figure S1). This effect was not different in the two genotypes. In addition, in female rats, the age × genotype interaction was statistically significant (Table 2) indicating that hypoactivity in the TgF344-AD rats was evident at 6 and 12 months of age, but at 18 months of age the activity curves were coincident for the two genotypes (Figures 3D–F and Supplementary Figure S1).

### Home Cage Activity

**FIGURE 4:**
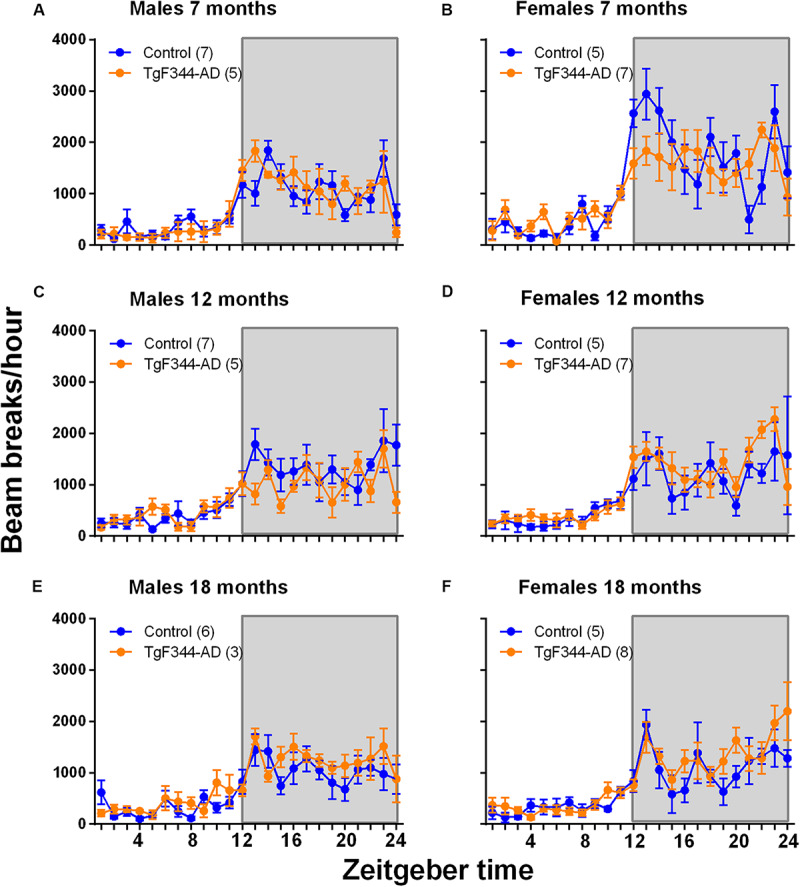
Home cage activity in a cross-sectional study in control and TgF344-AD male **(A,C,E)** and female **(B,D,F)** rats at 7 **(A,B)**, 12 **(C,D)**, and 18 **(E,F)** months of age. Each point represents the mean ± SEM for the number of rats indicated in parentheses. The abscissa represents 1-h epochs across time with epoch 1 representing 6 am–7 am; lights were on at 6 am and lights were off at 6 pm, as indicated by the shaded area. In males, only the main effect of epoch was statistically significant, indicating regardless of age or genotype, activity varies with epoch. In females, the age × epoch interaction was statistically significant indicating that regardless of genotype, younger rats were more active compared with the 12- and 18-month old animals; effects were statistically significant at 8th, 11th, 12th, and 20th epochs. Younger rats were more active than 18-month old rats at the 18th epoch.

We used a home-cage monitoring system to assess home cage activity in a cross-sectional analysis. In males, we found no statistically significant interactions or main effects of genotype or age. Only the main effect of epoch was statistically significant reflecting a normal circadian rhythm of activity (Table 1 and Figures 4A,C,E). In females, only the age × epoch interaction was statistically significant indicating that regardless of genotype, activity was higher in the younger animals compared with either the 12- and 18-months old groups at epochs 8, 11, 12, and 20. Activity was higher in the younger animals compared with only the 18-month old group at epoch 18 (Table 2 and Figures 4B,D,F). These epochs are in both the active and inactive phases.

### Immunohistochemistry

**FIGURE 5:**
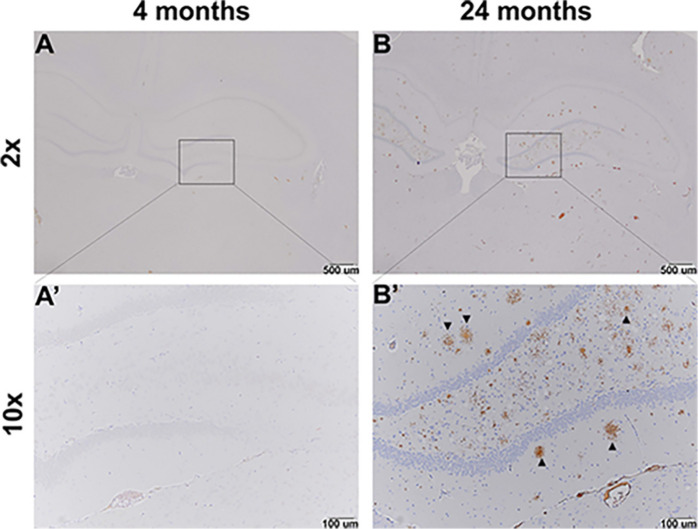
Representative immunohistochemistry images of amyloid-β in the hippocampus of TgF344-AD animals. Amyloid-β positive plaques are not evident in images from a 4-month-old rat **(A,A’)**. Whereas amyloid-β positive plaques are visible (arrows) in images from a 24-month-old rat **(B,B’)**.

**FIGURE 6:**
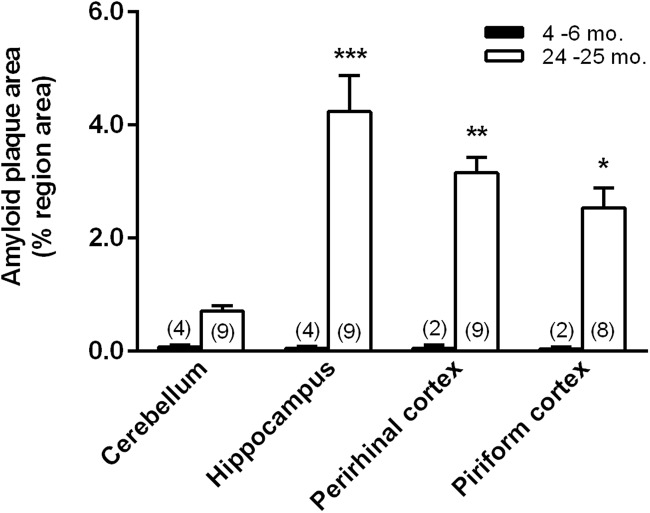
Amyloid-β positive plaque areas as a percent of region area in the field of view in 4–6 and 24–25 months old TgF344-AD rats in cerebellum, hippocampus, perirhinal cortex, and piriform cortex. We had good sections from only one TgF344-AD rat at 12 months of age, so these data are not shown. The rat analyzed at 12 months of age was female and values for cerebellum, hippocampus, and perirhinal cortex were 0.33, 1.76, and 2.53%, respectively. These values were intermediate between the values in the 4–6 and the 24–25 month rats. Bars represent means ± SEM for the number of animals indicated in parentheses. We compared male (4) and female (5) values at 24–25 months of age by means of unpaired *t*-tests, and we found no significant sex differences in any region (cerebellum, *p* = 0.373; hippocampus, *p* = 0.730; perirhinal cortex, *p* = 0.674; piriform cortex, *p* = 0.477). As a consequence, further analyses were performed with males and females at each age combined. Data were analyzed by means of ANOVA with age and region as between subject variables. Main effects of both age (*F*_1,39_ = 51.25, *p* < 0.0001) and region (*F*_3,39_ = 5.459; *p* = 0.0031) were statistically significant. The age × region interaction was also statistically significant (*F*_3,39_ = 5.557; *p* = 0.003) so we compared the two ages in each region by means of Sidak’s corrected *t*-tests. Levels of statistical significance are denoted as follows: *, 0.01 ≤ *p* ≤ 0.05; **, 0.001 ≤ *p* ≤ 0.01; ***, *p* ≤ 0.0001.

The presence of amyloid-β in brain was analyzed in a separate series of rats by means of immunohistochemistry (Figure 5). Fourteen TgF344-AD animals were analyzed. We counted amyloid-β deposits (plaques) and measured the area occupied by plaques in four 4–6 months old (three females, one male), one 12 months old (one female, zero males), and nine 24–25 months old (five females, four males) TgF344-AD rats (Figure 6). We expressed results as plaque area as a percent of region area in the section. We analyzed piriform cortex, perirhinal cortex, hippocampus, and cerebellum. Plaque area was very low at 4–6 months of age and increased significantly in hippocampus (82 fold), perirhinal cortex (63 fold), and piriform cortex (73 fold) at 24–25 months of age. In a single female TgF344AD rat at 12 months of age values were intermediate between the young and aged groups. We compared male (4) and female (5) values at 24–25 months of age by means of unpaired *t*-tests, and we found no significant sex differences in any region (cerebellum, *p* = 0.373; hippocampus, *p* = 0.730; perirhinal cortex, *p* = 0.674; piriform cortex, *p* = 0.477), therefore, sexes were combined for the analysis of age.

## Discussion

Results of our present study of the TgF344-AD transgenic rat confirm an age-dependent and region-dependent accumulation of amyloid-β plaques and a deficit in performance on a test of learning and memory. Female TgF344-AD rats at 6 and 12 months of age were hypoactive in the open field compared to controls. Our data also show evidence of hyposmia in maleTgF344-AD rats regardless of age and normal rest-activity rhythms at all three ages for both genotypes. These changes further elaborate the behavioral phenotype of this rodent model of AD.

At the outset we designed our study of the TgF344-AD rat model to include both male and female rats and to analyze the data from both sexes together. This was based on the initial report of this model in which no sex differences were found (Cohen et al., 2013). Our study addressed different phenotypes from the original study, however, and with respect to some of these phenotypes, we see sex differences. Male and female TgF344-AD animals appear to differ with respect to the buried food task. These results will be discussed below. Analysis of these data for each sex separately may leave our study somewhat underpowered, but analysis of the data from both males and females grouped together may lack validity given the presence of sex differences. Despite these limitations, our results demonstrate behavioral phenotypes in TgF344-AD rats. Subsequent studies investigating AD models should consider the likelihood of sex-differences at the outset.

Our study confirms and expands on the results reported previously on amyloid-β deposition in brain in the TgF344-AD model (Cohen et al., 2013). In the previous study amyloid-β deposition was observed in rats at 16 months of age in cingulate cortex and hippocampus and increased in density in 26 months old rats. Our results reveal some regional selectivity in brain regions affected. Three brain regions were particularly affected: hippocampus, piriform cortex, and perirhinal cortex. In these regions, plaque counts were very high in rats at 24–25 months of age. In view of the fact that deficiencies in spatial cognition are a primary symptom of AD, it is interesting that regions of the brain in which plaque counts are particularly affected in this rat model include regions of the navigation network (hippocampus and perirhinal cortex) (Pengas et al., 2012; Miller et al., 2014; Mitchell et al., 2018). The effects seen in piriform cortex are of interest in light of the involvement of piriform cortex in identification of odors and the deficiencies in olfaction in the rat model and in human subjects.

The learning and memory impairment confirms previous results in which the Barnes maze and novel object recognition task were used for assessment (Cohen et al., 2013). They also align with data from these rats using a Morris Water Maze (Berkowitz et al., 2018). Taken together, results indicate that these behavioral impairments are correlated with the histological abnormalities and neurodegeneration originally reported (Cohen et al., 2013).

We did not see hyperactivity in the open field in either 12- or 18-month old TgF344-AD rats as reported previously (Cohen et al., 2013). Higher activity in the first few epochs of the open-field as seen in the younger rats of both genotypes is indicative of an increased responsiveness to the novel environment. One possibility is that responsiveness to the open field declined with age because the rats remembered the open field arena from the prior test at 6 months. Our data indicate that 6- and 12-month old femaleTgF344-AD rats were hypoactive in the open field compared with controls. Hypoactivity in the younger TgF344-AD rats may also be apparent in the home cage activity record. Whereas rest-activity rhythms follow the typical active/inactive cycle in both genotypes, in females activity levels are higher in the younger rats. It is important to note that we did not control for the estrous cycle in the females and do not know what phase of the cycle the animals were in during the course of the experiment. It has been shown that the estrous cycle can affect activity levels of female rats (Finger, 1969).

One of the questions we set out to address with the buried food task was the development of a potential hyposmia phenotype. Our data are suggestive of an early appearing hyposmia phenotype in males, supporting the notion that hyposmia may be an early phenotype in AD and a possible clinical predictor (Fioretti et al., 2011). In females, hyposmia is not apparent until 18 months of age after we began noting differences in learning and memory and after amyloid-β plaques were apparent. Our data from the female rats are more consistent with the hypothesis that differences in odor detection (different from olfactory identification) are not present until later stages of the disorder (Serby et al., 1991; Xu et al., 2014). High levels of variability and limited numbers of rats per group prevent firm conclusions from these studies.

## Conclusion

The results of this study highlight the timing of changes in important behaviors in the TgF344-AD transgenic rat and suggest that some of these may be sex-dependent. Our results suggest a correlation between cognitive impairment and amyloid-β plaque formation. Hyposmia characterized TgF344-AD animals in general, and this phenotype appeared to be stronger in young adult males, whereas hypoactivity was evident in young TgF344-AD females. Some of these behaviors may represent early markers of AD progression and should be considered in future studies for early diagnosis of AD. We recommend that subsequent studies of AD in both animal models and in human subjects should further investigate sex-differences in AD progression and pathology.

## Data Availability Statement

The raw data supporting the conclusions of this article will be made available by the authors, without undue reservation, to any qualified researcher.

## Ethics Statement

The animal study was reviewed and approved by the NIMH Animal Care and Use Committee.

## Author Contributions

RS helped design the study, carried out the home cage activity studies and the analysis of the IHC results, analyzed the data, interpreted results, and wrote the manuscript. SC made pictures for the IHC studies, analyzed the data, and helped to writing the manuscript. LK designed the study and carried out the behavioral experiments. PZ carried out the IHC studies. RC interpreted results and edited the manuscript. CS designed the study, analyzed the data, interpreted results, and wrote the manuscript.

## Conflict of Interest

The authors declare that the research was conducted in the absence of any commercial or financial relationships that could be construed as a potential conflict of interest.
