# Supplementary material for: Behavioral Phenotype in the TgF344-AD Rat Model of Alzheimer’s Disease
Source: Front Neurosci. 2020 Jun 16;14:601. doi: 10.3389/fnins.2020.00601 (PMC7308710; doi:10.3389/fnins.2020.00601)
Supplement: Supplementary file 1 [file Data_Sheet_1.docx]

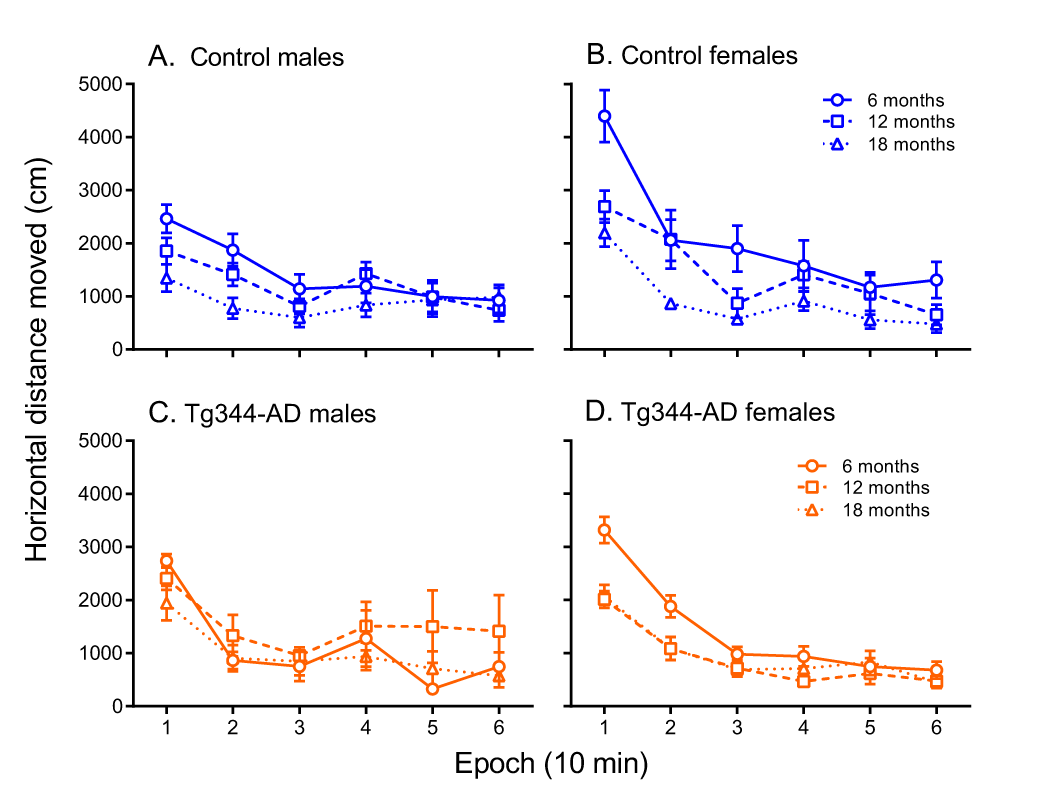


Supplemental Figure 1: Open field activity in male control (A), female control (B), male TgF344-AD (C), and female TgF344-AD (D) rats at 6, 12, and 18 months of age. Each point is the mean ± SEM in 9 male controls, 6 female controls, 5 male TgF344-AD, and 15 female TgF344-AD rats. For males, only the main effect of epoch was statistically significant (p < 0.001) indicating habituation over the 60 min testing period regardless of either age or genotype. For females, the age x epoch (p < 0.001) interaction was statistically significant indicating that regardless of genotype habituation decreased with increasing age likely due to decreased responsivity to the environment in the 12- and 18-month old animals. Moreover, in females, the genotype x age (p=0.013) interaction was statistically significant indicating that in the 6 and 12 month old groups, AD rats were hypoactive compared with controls; activity levels were similar in the two genotypes at 18 months of age.
